# Alpha 1 Antitrypsin Suppresses Autoantibody Production and Cellular Autoimmunity in Chronic Graft-Versus-Host Disease (cGVHD) in a Lupus Mouse Model

**DOI:** 10.3390/biom16030371

**Published:** 2026-03-01

**Authors:** Ahmed S. Elshikha, Georges Abboud, Jordan Stokes, Carolin Arnold, Nathalie Kanda, Laurence Morel, Sihong Song

**Affiliations:** 1 Departments of Pharmaceutics, University of Florida College of Pharmacy, Gainesville, FL 32610, USA; ahmedsamirelshikha85@gmail.com (A.S.E.); jordan.stokes@ufl.edu (J.S.); carolin.arnold25@web.de (C.A.); 2 Department of Pathology, Immunology and Laboratory Medicine, University of Florida College of Medicine, Gainesville, FL 32610, USA; georgeabboud117@gmail.com (G.A.); nathaliekanda@gmail.com (N.K.)

**Keywords:** human alpha1 antitrypsin (hAAT), systemic lupus erythematosus (SLE), chronic graft versus-host disease (cGVHD), autoimmunity, autoantibodies

## Abstract

Systemic lupus erythematosus (SLE) is a severe autoimmune disease that is challenging to treat due to poor understanding of its pathogenesis and etiology. Clearly understanding and dissecting the therapeutic effects of potential treatment in animal models are important. It has been shown that human alpha-1 antitrypsin (hAAT) holds therapeutic potential for the treatment of autoimmune diseases including lupus. However, the mechanism underlying its protective effect requires further investigation. In the present study, we used a chronic graft-versus-host disease-induced lupus mouse model to test the effect of hAAT on lupus development. We performed adoptive transfer of MHC I-aβ mismatched bm12 splenocytes into hAAT transgenic mice and showed that hAAT significantly blocked the production of anti-dsDNA IgG autoantibodies. Mechanistically, hAAT inhibited T cell activation and proliferation, including that of effector memory T (Tem) and T follicular helper (Tfh) cells. In addition, hAAT suppressed germinal center formation and functions. These results advanced the current understanding of hAAT functions and provide a new insight for the treatment of SLE.

## 1. Introduction

Systemic lupus erythematosus (SLE) is a severe and complex systemic autoimmune disease. In this condition, the over-reactive immune system leads to tissue damage in multiple organs including the joints, skin, brain, lungs, kidneys, and blood vessels. Mounting evidence shows that DCs and IFN-I play critical roles for initiating, developing and maintaining lupus disease [1]. Plasmacytoid dendritic cells (pDCs), which link innate and adaptive immunity, are a major cell source that produce IFN-I in response to autoantigens in the form of immune complexes. IFN-I can activate monocytes or conventional DCs (cDCs) to produce BAFF, a key factor that stimulates B cells to produce autoantibodies. We have shown that cytokines produced by activated cDCs from lupus mice enhance B cell proliferation and antibody secretion, and suppress regulatory T cell (Treg) differentiation and function [2]. In addition, activated DCs can present autoantigens and induce T cell differentiation by up-regulating MHCII and costimulatory molecules, which allow the direct modulation of the adaptive immune response. Activated pDCs can also indirectly impact T cells by producing inflammatory cytokines (e.g., TGF-β, TNF-α, IFN-I). The clinical heterogeneity and the pathological complicity of SLE makes it difficult to treat this disease [3,4]. Treatment with nonsteroidal anti-inflammatory drugs (NSAIDs), antimalarial drugs, corticosteroids and immune suppressants can control the symptoms and reduce tissue damage. However, these drugs have many debilitating side effects. Recent studies have shown that therapeutic strategies for resetting or reprogramming the immune system hold great potential for the treatment of this disease [5]. While engineered cell therapies (ECTs) are a powerful example, the development of an effective and safe therapy that can reset the immune system for the treatment of SLE is needed. As the disease is complex, testing therapeutic strategies in spontaneous and induced animal models of lupus are an important to assess the advantages or limitations of the therapy [6]. Among these models, the chronic graft-versus-disease (cGVHD)-induced model is unique as it mirrors common components of SLE in patients, including high levels of autoantibodies and a high frequency of T follicular helper cells (Tfh) and geminal center (GC) B cells, and it allows the use of transgenic mice on a C57BL/6 [7].

Human alpha-1 antitrypsin (hAAT), a 52 kD glycoprotein in circulation, is a member of the serin proteinase inhibitor (SERPIN) super-family. As a serum protein mainly produced from the liver, hAAT has anti-inflammatory and immune-regulatory functions. It can inhibit multiple enzymes including neutrophils elastase (NE), cathepsin K and caspase 3, and protect tissues from damage [8,9]. In addition, hAAT suppresses the production of pro-inflammatory cytokines (TNFα, IL-6, IL-1β, IL-8) and enhances the secretion of the anti-inflammatory cytokine IL-10 [10,11,12]. Mechanistic studies have shown that hAAT can directly interact with cell-surface receptors (TNFR1 and TNFR2) and regulate target cell gene expression [13]. We have shown that hAAT can also enter cells and interact with cellular enzymes [14] and reduce NF-kB activity [15]. Increasing evidence indicates that hAAT is a multifunctional protein that plays important roles in many biological processes, including modulating the immune system. Previous studies have shown hAAT has a therapeutic potential for the treatment of type 1 diabetes (T1D) [14,16], rheumatoid arthritis (RA) [17], SLE [18], stroke [19], hypertension [20] as well as bone loss [8,21], and it has antiaging effects [15]. However, the mechanism underlying its protective effect requires further investigation. We have recently shown that hAAT inhibits endosomal TLR signaling pathways by inhibiting TLR activation, the proteolytic process in the endosome [22]. In this study we used our recently developed hAAT transgenic mouse lines to test the effect of hAAT on cGVHD-induced lupus development.

## 2. Material and Methods

### 2.1. Animals

C57BL/6 (B6) and B6(C)-H2-Ab1bm12/KhEgJ (bm12) male mice were purchased at 8–12 weeks old from Jackson Laboratory (Bar Harbor, ME, USA). To test the effect of hAAT and mouse AAT on the development of lupus, we used the following mouse lines, which either express or lack hAAT or mouse AAT. Mouse-AAT knockout (mAAT-Ko) mice generated by Dr. Christian Mueller at the University of Massachusetts [23] were produced at the University of Florida. The hAAT-Tg mice with a B6 background were developed and maintained at the University of Florida [24]. The hAAT-Tg/Ko mice were generated by crossbreeding mAAT-Ko with hAAT-Tg mice. The heterozygous mice were subsequently crossbred to obtain hAAT-Tg^+^ mAAT^−^ (hAAT-Tg/Ko) mice. We identified hAAT-TG/Ko mice using hAAT-specific and mouse-AAT-specific ELISAs. All mice were housed in specific pathogen-free (SPF) conditions. For the induction of the lupus phenotypes, we employed cGVHD induction as previously described [7]. Briefly, 8- to 12-week-old B6 (as a control), hAAT-Tg, mAAT-Ko or hAAT-Tg/Ko male mice were i.p. injected with 60 to 70 × 10^6^ spleen cells from bm12 male mice. The number of animals per group was determined based on previous studies with this model demonstrating sufficient power to detect biologically meaningful differences in these immune populations [25]. We sacrificed all mice at 3 weeks after the cell transfers. All experiments were conducted according to protocols approved by the institutional animal care and use committee (IACUC) (UF-IACUC numbers: 20197848 approved on 5 June 2019 and 202200000256 approved on 13 June 2022) at the University of Florida.

### 2.2. Flow Cytometry

The spleens were processed as previously described [26]. The splenocytes (1 × 10^6^ cells) from recipient mice were blocked on ice with an Fc receptor blocker, anti-CD16/CD32. Then, cells were stained with the following FITC-, PE-, PECy7-, PB-, BV421-, APC-, PerCP, BV711-, V500-, PerCP eF710-, AF647, and APC R700- monoclonal antibodies to mouse CD4 (GK1.5), CD44 (IM7), CD62L (MEL-14), ICOS (7E.17G9), PD-1 (J43), Foxp3 (FJK-16S), Ki-67 (SolA15), BCL-6 (K112-91), BCL-2 (BCL/10C4), CD19 (6D5), B220 (RA3-6B2), CD138 (281-2), CD95 (Jo2), GL-7 (GL-7), and IgD (217-170). All antibodies were obtained from BD Biosciences (San Diego, CA, USA), Biolegend (San Diego, CA, USA), or eBioscience (San Diego, CA, USA). Stained cells were acquired using LSRFortessa from BD Biosciences (San Diego, CA, USA). Dead cells were excluded with fixable viability dye (LIVE/DEAD™ Fixable Yellow Dead Cell Stain Kit; Thermo Fisher Scientific, Waltham, MA, USA). Intracellular staining was performed with a fixation/permeabilization kit from eBioscience (San Diego, CA, USA). All samples were acquired on an LSRFortessa flow cytometer from BD Biosciences (San Diego, CA, USA) and analyzed with FlowJo V10 software (Tree Star, Woodburn, OR, USA). Briefly, dead cells were removed using a fixable viability dye and doublets were excluded by singlet gating. From the live singlet lymphocytes, the CD4^+^ T cells were identified and subdivided as follows: effector memory T cells (Tem): CD4^+^CD44^+^CD62L^−^; T follicular helper (Tfh) cells: CD4^+^CD44^+^CD62L^−^PD-1^+^BCL-6^+^; and proliferating subsets were defined by Ki-67 expression within each respective parent gate. B cells were defined as B220^+^CD19^+^ and subdivided as: germinal center (GC) B cells: B220^+^CD19^+^GL-7^+^CD95^+^; and proliferating GC B cells were identified as Ki-67^+^ within the GC gate. Plasma cells were defined as IgD^−^CD138^+^ cells, and proliferating plasma cells were identified as Ki-67^+^ within this gate. These definitions are now linked to the relevant figure panels. All flow cytometric acquisition and downstream analyses were performed in a blinded fashion with respect to treatment group.

### 2.3. Autoantibody Measurement

Blood samples were collected 3 weeks after splenocyte injection and the serum anti-dsDNA IgG autoantibodies were detected by ELISA with the serum diluted 1:100 as previously described [27].

### 2.4. Detection of Serum hAAT and Mouse AAT

Serum hAAT levels in the B6 (control), mAAT-Ko, hAAT-Tg/Ko, and hAAT-Tg mice were detected by hAAT-specific ELISA as previously described [28]. Briefly, purified hAAT (Athens Research Technology Inc., Athens, GA, USA) was used as the standard. Goat-anti-hAAT antibodies were used as the primary antibody. Rabbit anti-hAAT and peroxide-conjugated goat anti-rabbit IgG were used as secondary and tertiary antibodies. The mouse-AAT levels in all mice were detected by ELISA as previously described [29]. Briefly, pooled B6 (adult male) mouse serum was used as a standard, in which the mouse-AAT concentration was defined as one relative unit. The mouse serum samples were diluted and incubated in a microtiter plate (Immulon 4, Dynex Technologies, Chantilly, VA, USA) in Voller’s buffer overnight at 4 °C. Plates were blocked with 3% bovine serum albumin for 1 h at 37 °C. Then, samples were incubated for 1 h at 37 °C. The chicken anti-mouse alpha 1-antitrypsin polyclonal antibody (1:1600 dilution, MyBioSource, San Diego, CA, USA) and HRP-conjugated goat anti-chicken-IgG antibody (1:5000 dilution, ThermoScientific, Waltham, MA, USA) were added and incubated for 1 h at 37 °C. The plates were washed with PBS-Tween 20 (Sigma-Aldrich, St. Louis, MO, USA) between reactions. After adding the substrate (O-Phenyldiamine, Sigma-Aldrich, St. Louis, MO, USA), the plates were read at 490 nm on an MRX microplate reader (Dynex Technologies, Chantilly, VA, USA). The OD reading of each sample was used to calculate the relative unit based on the standard curve, in which the pooled B6 (adult male) sera was used as a standard. The normal mouse-AAT concentration was defined as one relative unit.

### 2.5. Statistics

Data analysis was performed using Graphpad Prism 9.0 software. Differences between groups were evaluated by one-way ANOVA with correction for multiple tests, or *t* tests, as indicated in the text. The results were expressed as mean ± standard deviation. The thresholds for statistical significance were set at *: *p* < 0.05, **: *p* < 0.01, ***: *p* < 0.001 and ****: *p* < 0.0001.

## 3. Results

### 3.1. Transgenic Expression of hAAT Inhibits Autoantibody Production in the cGVHD-Induced Lupus Mouse Model

**Figure 1:**
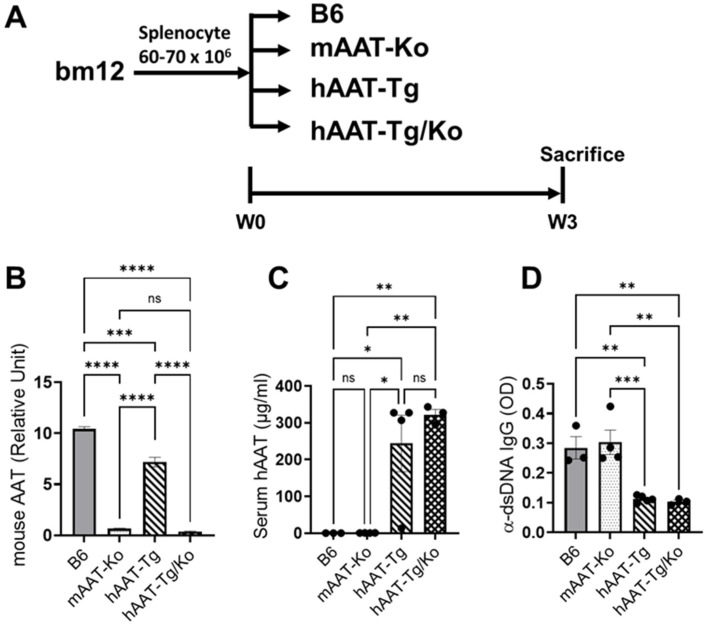
Animal models, experiment design and autoantibody production. (**A**) Experimental design. B6 (n = 3), mAAT-Ko (n = 4), hAAT-Tg (n = 4) and hAAT-Tg/Ko (n = 3). (**B**) Mouse-AAT levels detected by mouse-AAT specific ELISA. (**C**) Human-AAT levels detected by hAAT-specific ELISA. (**D**) Anti-dsDNA IgG autoantibody levels detected by ELISA. * *p* < 0.05, ** *p* < 0.01, *** *p* < 0.001, **** *p* < 0.0001. ns, not significant *p* > 0.05.

The adoptive transfer of MHC I-aβ mismatched bm12 spleen cells into B6 mice results in an SLE-like syndrome characterized by autoantibody production and immunopathology [30,31]. We have previously shown that hAAT treatment, administered either as a protein or gene therapy, inhibited autoantibody production and ameliorated disease development in spontaneous lupus-prone mice [18]. In this study, we took advantage of the bm12-induced model of lupus as it allows rapid identification of the effect of a particular gene on lupus development. To evaluate the effect of hAAT and mouse AAT (mAAT) on the development of lupus in this model, we used four strains of mice; B6 mice (disease model control), mouse-AAT knockout mice (mAAT-Ko), human-AAT transgenic mcie (hAAT-Tg), and human-AAT-Tg mice that lack mouse AAT (hAAT-Tg/Ko) (Figure 1A). As expected, serum levels of mouse AAT were detected in both the B6 and hAAT-Tg mice, but not in the mAAT-Ko and hAAT-Tg/Ko mice (Figure 1B). Conversely, serum levels of hAAT were detected in the hAAT-Tg and hAAT-Tg/Ko mice, but not in the B6 and mAAT-Ko mice (Figure 1C). All mice received splenocytes from bm12 mice (Figure 1A). Three weeks after bm12 cell transfer, the anti-dsDNA IgG autoantibody levels in the B6 and mAAT-Ko mice were clearly induced to detectible levels, indicating successful lupus induction. Importantly, autoantibody levels in the hAAT-Tg and hAAT-Tg/Ko groups were significantly lower than those in the B6 and mAAT-Ko groups (Figure 1D). These results indicate that the presence of hAAT inhibited autoantibody production, whereas mAAT apparently has no significant effect on the production of autoantibodies under these conditions.

### 3.2. hAAT Inhibits T Cell Activation and Proliferation

**Figure 2:**
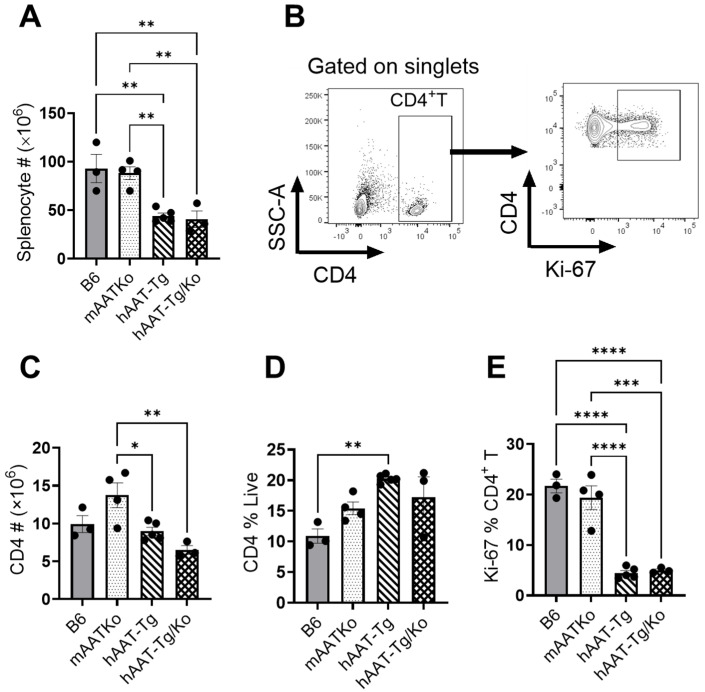
The effect of hAAT on T cells. (**A**) Total number of splenocytes. (**B**) Gating strategy for CD4+ T cells. (**C**) Number of CD4+ T cells. (**D**) Percentage of CD4+ T cells. (**E**) Proliferating CD4+ T cells. B6 (n = 3); mAAT-KO (n = 4); hAAT-Tg (n = 4); hAAT-Tg/Ko (n = 3). * *p* < 0.05, ** *p* < 0.01, *** *p* < 0.001, **** *p* < 0.0001.

In the cGVHD-induced lupus model, the interaction of donor bm12 CD4+ T cells with MHC class II on the B cell surface of the B6 host results in the proliferation of Tfh cells, GC B cells, and plasma cells [25,32]. To examine the effect of hAAT on these populations, we next evaluated immune cell subsets by flow cytometry. Three weeks after bm12 splenocyte transfer, the total splenocyte numbers were significantly lower in both the hAAT-Tg and hAAT-Tg/Ko groups compared with the the B6 and mAAT-Ko groups (Figure 2A). Flow cytometry analysis showed that the total number of CD4+ T cells in the mAAT-Ko group was significantly higher than that in the hAAT-Tg and hAAT-Tg/Ko groups (Figure 2B,C). Although the frequency of total CD4+ T cells was significantly higher in hAAT-Tg mice than in the B6 mice (Figure 2D), the frequency of proliferating CD4^+^ Ki-67^+^ T cells was significantly lower in both the hAAT-Tg and hAAT-Tg/Ko groups compared with the B6 and mAAT-Ko groups (Figure 2E).

### 3.3. Inhibitory Effect of hAAT on T Effector Memory (Tem) Cells

**Figure 3:**
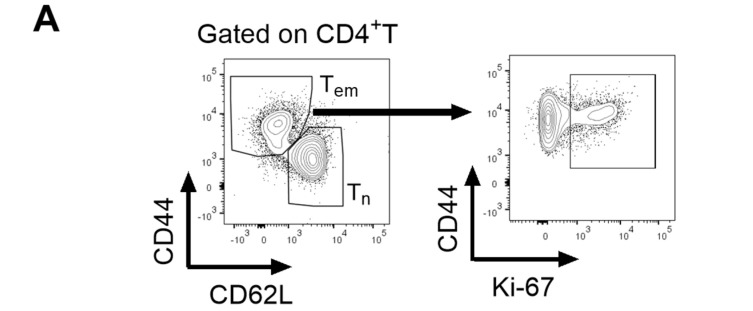

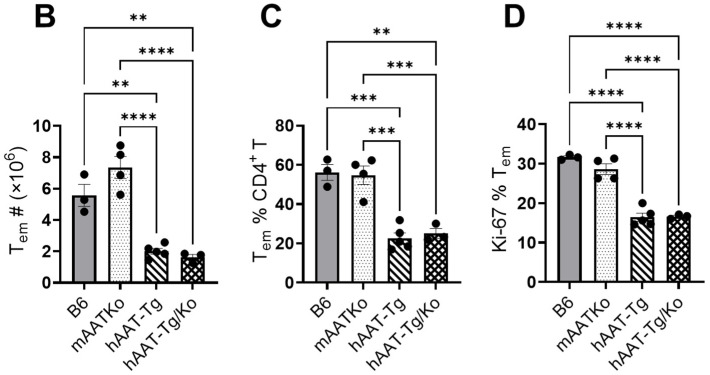
The effect of hAAT on Tem cells. (**A**) Gating strategy for T effector memory (Tem) cells. (**B**) Number of Tem cells. (**C**) Percentage of Tem cells. (**D**) Proliferating Tem cells. G. B6 (n = 3); mAAT-KO (n = 4); hAAT-Tg (n = 4); hAAT-Tg/Ko, (n = 3). ** *p* < 0.01, *** *p* < 0.001, **** *p* < 0.0001.

**Figure 4:**
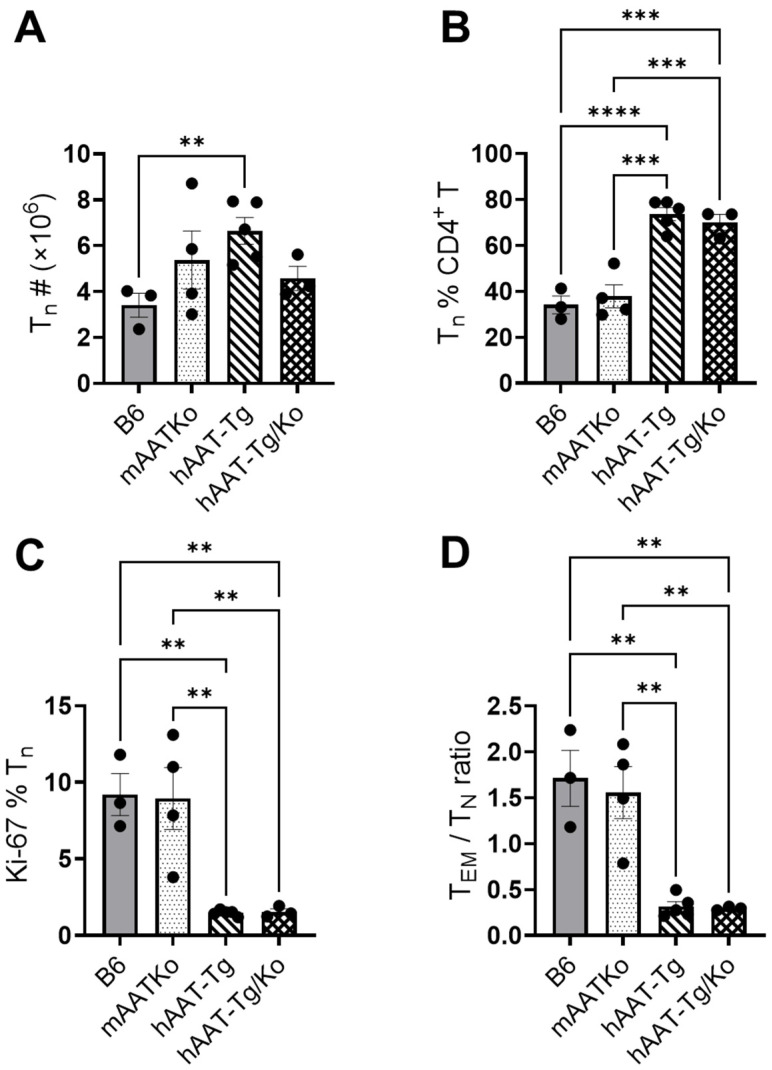
The effect of hAAT on Tn cells. (**A**) Number of Tn cells. (**B**) Percentage of Tn cells. (**C**) Percentage of Tn cells. (**D**) The ratio of Tem to Tn cells. B6 (n = 3); mAAT-KO (n = 4); hAAT-Tg (n = 4); hAAT-Tg/Ko, (n = 3). ** *p* < 0.01, *** *p* < 0.001, **** *p* < 0.0001.

We next investigated the effect of hAAT on T effector memory (Tem) cells. As shown in Figure 3, both the frequency and the total number of CD4+ Tem were significantly lower in the hAAT-Tg and hAAT-Tg/Ko mice compared with the B6 and mAAT-Ko groups. In addition, the proliferating Tem cells (Figure 3D) were significantly lower in the hAAT-Tg and hAAT-Tg/Ko mice than in the B6 and mAAT-Ko groups. We also observed that the frequency and numbers of CD4^+^ CD62L^+^-naive T cells (Tn) in the hAAT-Tg and hAAT-Tg/Ko groups were significantly higher compared with the B6 and mAAT-Ko mice (Figure 4A,B). Interestingly, Tn cell proliferation and the Tem/Tn ratio were significantly lower in the hAAT-positive groups than in the B6 and mAAT-Ko groups (Figure 4C,D). Together, these results demonstrate that hAAT exerts a significant inhibitory effect on Tem cell proliferation.

### 3.4. hAAT Expression Affects T Follicular Helper (Tfh) Cells

**Figure 5:**
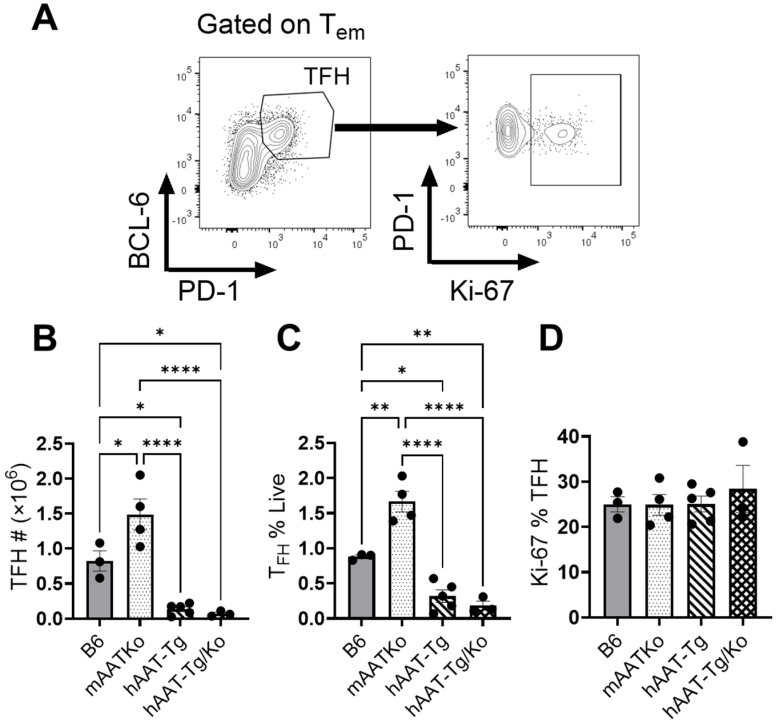
The effect of hAAT on TFH cells. (**A**) Gating strategy for T follicular helper (TFH) cells. (**B**) Number of TFH cells. (**C**) Percentage of TFH cells. (**D**) Proliferating TFH cells. B6 (n = 3); mAAT-KO (n = 4); hAAT-Tg (n = 4); hAAT-Tg/Ko, (n = 3). * *p* < 0.05, ** *p* < 0.01, **** *p* < 0.0001.

Because Tfh cells play an important role in lupus development, we investigated the effect of hAAT on Tfh cell populations. As shown in Figure 5, both the frequency and total numbers of Tfh cells were significantly lower in the hAAT-positive groups than in the B6 and mAAT-Ko groups (Figure 5A–C). However, hAAT has no effect on Tfh cell proliferation (Figure 5D). Together, these results clearly demonstrate that hAAT plays a critical role in inhibiting Tfh cells.

### 3.5. The Effect of hAAT on B Cells

**Figure 6:**
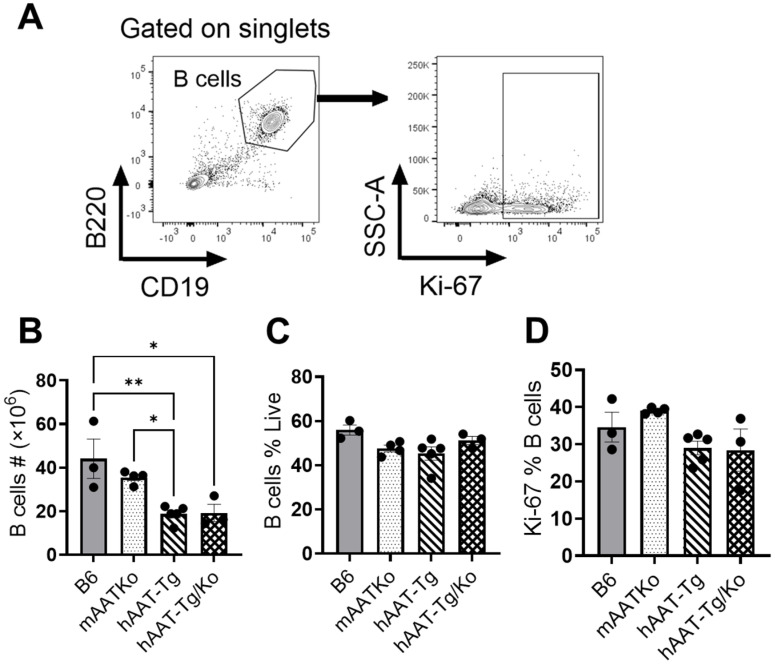
The effect of hAAT on B cells. (**A**) Gating strategy for B cells. (**B**) Number of B cells. (**C**) Percentage of B cells. (**D**) Proliferating B cells. B6 (n = 3); mAAT-KO (n = 4); hAAT-Tg (n = 4); hAAT-Tg/Ko, (n = 3). * *p* < 0.05, ** *p* < 0.01.

In the cGVHD model, B cells function as efficient antigen-presenting cells (APCs). During B–T cell interactions, the bm12 T cells help the host B cells, promoting clonal expansion, differentiation, and the survival of pathogenic T cells [31]. We therefore investigated the effect of hAAT on B cells. As shown in Figure 6, the total number of B cells was significantly lower in the hAAT-Tg and hAAT-Tg/Ko groups compared with the B6 and mAAT-Ko mice. However, hAAT expression did not affect B cell frequency and proliferation (Figure 6C,D).

### 3.6. hAAT Suppresses Germinal Center Formation and Function

**Figure 7:**
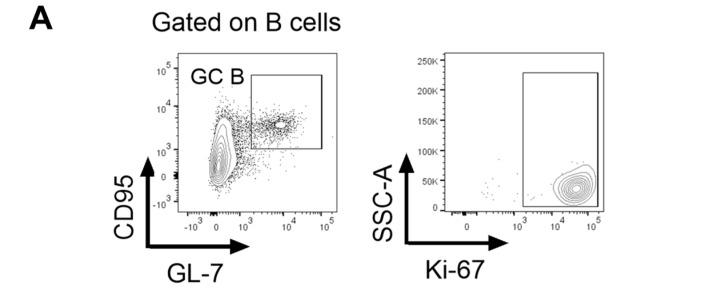

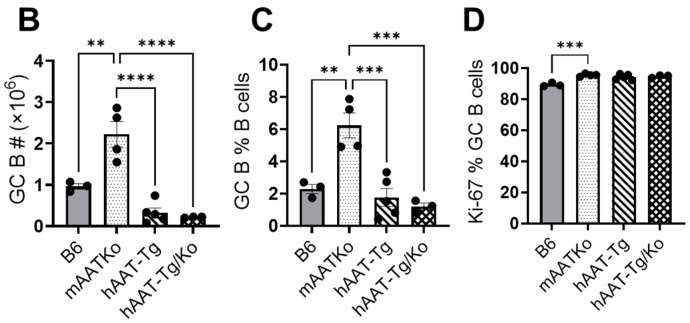
The effect of hAAT on germinal center (GC) B cells. (**A**) Gating strategy for GC B cells. (**B**) Number of GC B cells. (**C**) Percentage of GC B cells. (**D**) Proliferating GC B cells. B6 (n = 3); mAAT-KO (n = 4); hAAT-Tg (n = 4); hAAT-Tg/Ko, (n = 3). ** *p* < 0.01, *** *p* < 0.001, **** *p* < 0.0001.

**Figure 8:**
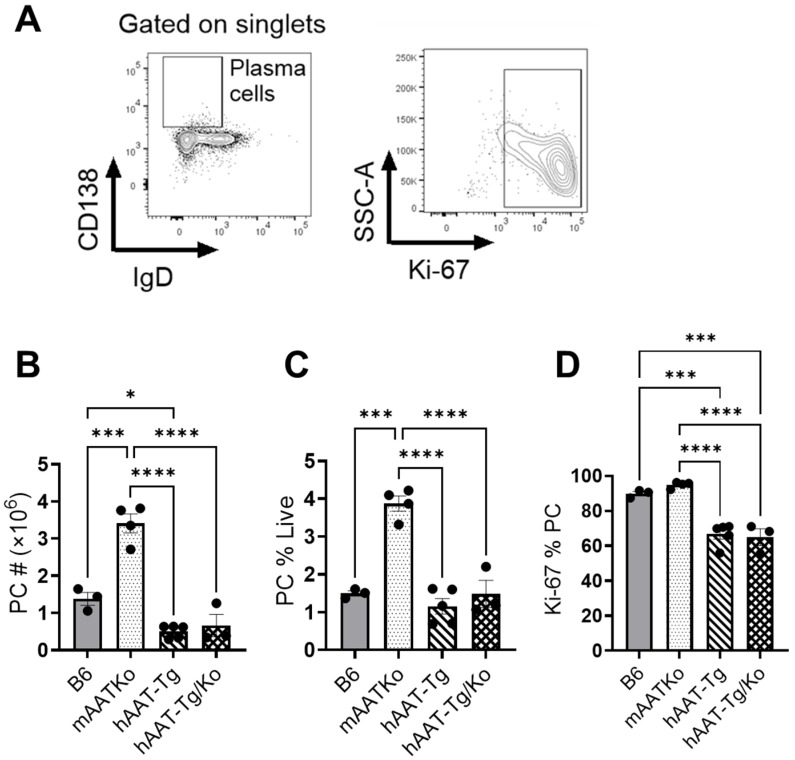
The effect of hAAT on plasma cells (PCs). (**A**) Gating strategy for PCs. (**B**) Number of PCs. (**C**) Percentage of PCs. (**D**) Proliferating PCs. B6 (n = 3); mAAT-KO (n = 4); hAAT-Tg (n = 4); hAAT-Tg/Ko, (n = 3). * *p* < 0.05, *** *p* < 0.001, **** *p* < 0.0001.

We next investigated the effect of hAAT on germinal center B cells. As shown in Figure 7, both the number and frequency of GC B cells were significantly higher in mAAT-Ko mice than in B6 mice, and these levels were significantly reduced in the hAAT-Tg and hAAT-Tg/Ko mice. In addition, GC B cell proliferation in the mAAT-Ko mice was also significantly higher than that in B6 mice (Figure 7D). These results indicate that deficiency of mouse AAT leads to an increase in GC B cells and hAAT expression can compensate for this deficiency. We also examined the effect of hAAT on plasma cells (PCs). As shown in Figure 8, both the total number and frequency of PCs in the mAAT-Ko group were significantly higher than that in the B6 group and the hAAT-positive groups. Moreover, PC proliferation was significantly lower in the hAAT-positive groups than in the mAAT-Ko and B6 groups. Taken together, these results demonstrate for the first time that the absence of mouse AAT may enhance GC B cell differentiation and proliferation, leading to increased plasma cells, whereas the presence of hAAT significantly inhibits the germinal center formation and function.

## 4. Discussion

Lupus is a life-threatening autoimmune disease that currently has no cure. We have previously shown that the hAAT gene and protein therapy effectively prevent lupus development in spontaneous lupus mouse models. However, the mechanisms by which hAAT functions remain to be investigated. In this study, we exploited a cGVHD-induced model and tested AAT functions in our recently developed hAAT-TG, AAT-TG/KO and AAT-KO mouse lines. We demonstrated that transgenic expression of hAAT blocked autoantibody production, which has a critical role in lupus development. Mechanistically, AAT inhibited T cell activation and proliferation and suppresses germinal center (GC) formation and function. Consistent with previous observations, these results advance our understanding of hAAT biology and provide new insights for developing therapies for the treatment of SLE in humans.

One of the questions for using hAAT to treat autoimmune diseases such as SLE is why patients need hAAT treatment, since most of them are not diagnosed as AAT-deficient (AATD, most commonly PiZZ mutation of hAAT gene). Although this issue requires further investigation, accumulating evidence supports the AAT insufficiency hypothesis [9]. The hAAT gene is up-regulated in response to inflammation, which explains why many patients with autoimmune diseases exhibit higher serum hAAT levels [33,34]. However, this up-regulation may not be sufficient for control of the disease for several reasons: (1) In pathological conditions, hAAT demand or consumption may increase [18]; (2) hAAT functions may be impaired in patients with autoimmune diseases [35]; and (3) recent studies discovered additional mutations in the *SERPINA1* gene, many of which can alter hAAT gene expression and the function, although their full impact remains unclear [36]. Because autoimmune diseases are characterized by heterogeneity, identifying patient sub-populations that may benefit most from hAAT therapy could lead to more effective treatments. Therefore, future studies should determine whether hAAT therapy is more effective in patients with impaired hAAT levels or functions.

Consistent with earlier reports, we observed that hAAT inhibited autoantibody production in this study [17,18]. The underlying mechanism is likely multifactorial. First, hAAT can interact with inflammatory mediators, thereby reducing autoantibody production [13,37]. Second, hAAT can block cell-surface and intracellular receptor functions (e.g., TNFR and TLR9), thereby indirectly affecting autoantibody production [13,22]. Third, hAAT can enter cells and inhibit cellular enzymes [14] and transcription factor activity [15]. Intriguingly, in this study we found that hAAT inhibited GC formation and functions, providing new insight into its mechanism of action. Future studies should investigate whether this effect extends to other antibody-producing responses, which would further support hAAT’s therapeutic potential in human diseases.

Testing hAAT in mouse models may be confounded by endogenous mouse AAT, raising a question of whether mouse AAT alters or enhances hAAT function. In mice, there are six genes (*Serpina1a-e*) encoding six different AAT isoforms. These genes, each about 11 kb in length, share highly conserved sequences (including introns) and are clustered on chromosome 12, spanning about 230 kb. This arrangement makes it difficult to create a mouse-AAT knockout (mAAT-Ko) model. Borel et al. used CRISPR/Cas9-mediated gene-editing technology and successfully created a mouse-AAT-Ko mouse model [23]. To test hAAT function without the effect of mouse AAT, we generated a hAAT-Tg/Ko mouse line by cross-breeding mAAT-Ko mice with hAAT-Tg mice. Using this new mouse model, we demonstrated that hAAT had a significant effect on lupus development. These results indicate that our hAAT-Tg/Ko mouse model may be useful for testing hAAT in other induced disease models.

Previous studies have shown that hAAT has therapeutic potential for GVHD. In animal models, the mechanisms underlying the protective effect of hAAT include altering the ratio of donor T effector to T-regulatory cells [38] and decreasing serum heparan sulfate (HS) and alloreactive T cell responses [39]. Treatment with hAAT-expressing mesenchymal stromal cells (MSCs) can also attenuate GVHD development in mouse models [40]. In humans, AAT plasma levels in donors are inversely correlated with the development of acute GVHD in recipients [41]. Clinical studies showed that hAAT treatment in patients with steroid-resistant acute graft-versus-host disease (SR-aGVHD) was safe and potentially effective [42,43]. However, the application of hAAT in lupus-related cGVHD models has not been investigated. Our results in this study extend the current understanding of hAAT’s functions and its potential applications.

This study has several limitations: (1) The sample sizes are relatively small because of the limited breeding colonies. Although the results clearly demonstrate that hAAT affects cGVHD-induced lupus markers, larger cohorts in future studies would provide greater statistical confidence. (2) We used male mice to evaluate hAAT function. While previous work has shown a protective effect in female mice, future studies with female hAAT-Tg and mAAT-Ko mice could determine whether sex differences modulate hAAT’s therapeutic efficacy in cGVHD-induced lupus. (3) Leveraging the cGVHD mouse model, this study focuses on hAAT’s effects on Tfh cells, GC B cells, plasma cells, and anti-nuclear autoantibody production. Subsequent research should elucidate the underlying mechanisms and investigate hAAT’s impact on related renal injuries.

## 5. Conclusions

In this study, we tested hAAT functions against cGVHD-induced lupus using recently developed hAAT-Tg, hAAT-Tg/Ko and mAAT-Ko mouse lines. We have shown that transgenic hAAT expression inhibits anti-dsDNA antibody production and the proliferation of T follicular helper (Tfh) cells and GC B cells. These findings are consistent with and extend the current knowledge of hAAT biology and suggest that the hAAT-Tg and AAT-Tg/Ko mouse models may be valuable tools for testing hAAT functions in other induced disease models.

## Data Availability

The original contributions presented in the study are included in the article, further inquiries can be directed to the corresponding authors.
